# Ultrasonication effects on graphene composites in neural cell cultures

**DOI:** 10.3389/fnmol.2022.992494

**Published:** 2022-09-16

**Authors:** Łucja Dybowska-Sarapuk, Weronika Sosnowicz, Anna Grzeczkowicz, Jakub Krzemiński, Małgorzata Jakubowska

**Affiliations:** ^1^Faculty of Mechatronics, The Institute of Metrology and Biomedical Engineering, Warsaw University of Technology, Warsaw, Poland; ^2^Centre for Advanced Materials and Technologies CEZAMAT, Warsaw, Poland; ^3^Nalecz Institute of Biocybernetics and Biomedical Engineering, Polish Academy of Sciences, Warsaw, Poland

**Keywords:** graphene nanoplatelets, surfactants, cell electrostimulation, sonication method, graphene substrates, spray-coating

## Abstract

Spinal cord injuries and neurodegenerative diseases, including Parkinson’s, Alzheimer’s, and traumatic brain injuries, remain challenging to treat. Nowadays, neural stem cell therapies excite high expectations within academia. The increasing demand for innovative solutions in regenerative medicine has drawn considerable attention to graphene materials. Due to unique properties, carbon materials are increasingly used as cellular scaffolds. They provide a biological microenvironment supporting cell adhesion and proliferation. The topography and mechanical properties of the graphene culture surface influence the forces exerted by the cells on their extracellular matrix. Which consequently affects the cell proliferation and differentiation. As a result, material properties such as stiffness, elasticity and mechanical strength play an important role in stem cells’ growth and life. The ink unification process is crucial while the layer homogeneity is essential for obtaining suitable surface for specific cell growth. Different ink unification processes were tested to achieve appropriate layer homogeneity and resistivity to successfully applied the GNPs layers in neural cell electrostimulation. The GNP coatings were then used to electrostimulate mouse NE-4C neural stem cells. In this study, the authors investigated how the stimulation voltage amplitude’s value affects cell behaviour, particularly the number of cells. Sinusoidal alternating current was used for stimulation. Three different values of stimulation voltage amplitude were investigated: 5, 10, and 15 V. It was noticed that a lower stimulation voltage amplitude had the most favourable effect on the stem cell count.

## Introduction

Stem cell therapies are gaining popularity due to their versatility, self-renewal, and multipotency (Ryu and Kim, 2013). Nervous stem cells are prime candidates for treating neurodegenerative diseases such as nerve cord injury, neuropathies, and traumatic brain injury (Lin et al., 2019). Therapeutic strategies for the peripheral nervous system (PNS) include pharmacotherapy, physiotherapy, and microsurgical procedures to bypass the ends of damaged nerves. Accelerated growth, increased number of nerve cells, and differentiation into a specialised local class and morphology affect the regeneration efficiency of the damaged nerves. The generation of an adequate number of synaptic connections is necessary to achieve electrophysiological functionalisation and information distribution (Valenzuela et al., 2007). There is no effective and reproducible therapeutic strategy to regenerate damaged nerves. Using graphene substrates and an electric field to stimulate cell proliferation and differentiation is an innovative approach that may provide an opportunity for accelerated regeneration of damaged nerve connections and spinal cord injuries.

The ideal material for neuronal stimulation should be characterised by low electrical impedance, sizeable electroactive surface area, and appropriate biomimetics (Krukiewicz et al., 2019). Hence, nanomaterials attract much attention (Gupta et al., 2019; Krukiewicz et al., 2019). Unique physicochemical properties, primarily high conductivity and the structure, which is similar to natural ECM, make carbon nanomaterials promising in the case of neuronal stimulation. Additionally, such substrate properties as elasticity, stiffness, roughness, wettability, and topology can regulate cell behaviour, adhesion, growth, and viability (Li et al., 2011; Chen et al., 2012; Menaa et al., 2015; Lin et al., 2019). Graphene materials significantly advance tissue engineering research with their unique mechanical, electrical, and optical properties (Gardin et al., 2016).

Creating a scaffold and a microenvironment similar to the ECM enables cells to adhere, proliferate and differentiate appropriately (Gupta et al., 2019), which are the primary roles of regenerative tissue processes. Cell adhesion to the biomimetic graphene scaffold mimicking the extracellular matrix stimulates the intracellular signalling pathway involved in the adhesion and migration of cells involved in neurogenesis (Lee et al., 2015). The forces exerted by cells on their extracellular matrix and the resulting activation and suppression of genes specific for cell proliferation depend on the mechanical properties and topography of the culture surface. Numerous scientific studies (Li et al., 2011; Ku et al., 2013; Guo et al., 2016; Eivazzadeh-Keihan et al., 2019) have demonstrated the efficacy of using graphene family materials for cellular stimulation of human mesenchymal stem cells (Lee et al., 2015), human fibroblasts (Dybowska-Sarapuk, 2019), osteoblasts (Aryaei et al., 2014), and neural cells (Li et al., 2011), among others. However, the complex molecular mechanisms underlying the self-renewal and differentiation of neural stem cells are not yet fully understood, and continued research on this topic is needed (Lin et al., 2019).

The existing solutions do not achieve the desired reproducible therapeutic effect, and there is a significant discrepancy between scientific reports (Huang et al., 2012; López-Dolado et al., 2015; Patel et al., 2015). Simultaneously, obtaining cytocompatible substrates with high conductivity to enhance cell proliferation is still a great challenge (Lee et al., 2011; Park et al., 2011; Sherrell et al., 2014). Fabricating polymer composites based on graphene materials is vital to ensure adequate particle dispersion. Homogeneous dispersion of particles allows for obtaining a composite with desired mechanical and electrical properties. The size and thickness of the particles and their interaction with the polymer matrix should also be considered when designing the composite (Papageorgiou et al., 2017; Cataldi et al., 2018). The choice of the mixed preparation method is also crucial and is selected according to the type and form of the composite. One of the most popular methods is solution mixing (ultrasound or temperature) (Singh et al., 2011; Papageorgiou et al., 2017). Rheological measurements of polymer composites are an effective tool to evaluate the usefulness of the material in given printing technology (Kim et al., 2010). The viscosity of the solution affects, among others, the efficiency of transmission of ultrasonic waves used during the sonication process responsible for breaking up agglomerates of functional phase particles and dispersing them in the polymer matrix (Zhang and Chen, 2019). Nguyen et al. (2007) note that the viscosity of nanofluids increases with the increasing particle percentage. They also report higher viscosity values of solutions produced with larger-size particles. A more significant concentration of particles in solution affects the internal shear stress of the fluid, resulting in increasing solution viscosity. In addition, such phenomena as particle agglomeration also increase nanofluid viscosity. Also, the type and properties of the ink components used, viscosity and tension of the matrix material and solvent significantly affect the rheology of the produced ink (Zhang and Chen, 2019).

Modifying the particle surface by attaching surfactants is a standard procedure to improve the homogeneity of solutions and counteract the agglomeration of hydrophobic particles. The homogeneous dispersion of graphene particles in solution and their efficient distribution in the polymer matrix can be achieved using ultrasound (Singh et al., 2011). The large specific surface area of graphene, which is an undoubted advantage in achieving high conductivity and modification of its surface, also triggers such undesirable phenomena as aggregation. One of the most effective methods to obtain homogeneously disperse particles in a polymer matrix is sonication using ultrasound. The quality of the process depends on such parameters as sonication time, ultrasound power, and amplitude. The type of the used solvent also brings a significant effect, as well as its temperature, which increases during the process itself (Zhang and Chen, 2019). Exposure to ultrasound could also cause the fragmentation of graphene flakes, and, as a result, affects the properties of the composite: mechanical strength and electrical conductivity (Si and Samulski, 2008; Liao et al., 2011; Zhang and Chen, 2019). This phenomenon could be significant in biomedical applications since flakes with smaller dimensions show more cell toxicity.

Due to the unique electrical properties of graphene, it is possible to combine two types of stimulation: material and electrical (Ku et al., 2013). The elucidation of the complex biological reactions occurring during the cell stimulation process and the ability of graphene materials to regulate cell behaviour is the subject of many scientific considerations (Balikov et al., 2016). To provide an effective and, at the same time, safe stimulation, the parameters of the electrical signal should be pre-set in such a way as to induce a physiological response and not cause damage to the tissue or the electrode itself (Krukiewicz et al., 2019). In addition, the resistance of the culture substrate should be as low as possible to apply a signal with a lower electrical potential (Heo et al., 2011; Krukiewicz et al., 2019). These assumptions find confirmation in the results of experiments by several researchers, including Huang et al. (2019), Heo et al. (2011), Uz et al. (2020). The relevant literature review features reports on the effectiveness of weak electric field electrostimulation with voltage values ranging from 4.5 mV (Heo et al., 2011), 10 mV (Huang et al., 2019) and 25, 50, and 100 mV (Das et al., 2017; Huang et al., 2019; Uz et al., 2020). However, in some cases, despite achieving an increased rate of cell count, electrostimulation only slightly affected the differentiation of cells on the graphene substrate and thus the formation of cell networks (Heo et al., 2011; Huang et al., 2019). Heo et al. (2011) also report observing no beneficial effect of applied electrostimulation of cells on graphene substrates (on PET film) on cell viability, with insignificant differences between cells from control cultures on glass culture dishes. Stimulation parameters such as period, frequency, and electrical voltage value need to be tailored to the specific cell group (Meng, 2014). Uz et al. (2020) and Sherrell et al. (2014) report different trends of cell differentiation depending on the stimulation voltage value. The authors observed a greater differentiation of cell populations into cells with a neural phenotype when stimulations with lower voltage values were applied. The results of studies conducted by several other researchers support the hypothesis that electrical stimulation using graphene-based substrates and appropriate modulation of electrical parameters enhances the mechanisms of cell differentiation towards neural phenotypes. However, the low percentage of differentiated cells (10% of the population) remains a significant problem (Uz et al., 2020).

The growing interest in graphene materials raises questions about their short- and long-term cytotoxicity. The dimensions, shape, number of layers, as well as the purity of the material and its hydrophilicity can affect the behaviour and physiology of cells, and thus determine its cytocompatibility and regenerative potential (Tang et al., 2013). Cytotoxicity of graphene materials requires analysis of its effects on organisms *in vitro* and *in vivo*, studying the degree of particle accumulation in tissues or blood vessels (Mansuriya and Altintas, 2020).

This article presents technology for fabricating graphene substrates and a strategy for stimulating mouse neural stem cells using external electrical stimulation. The improved graphene ink fabrication technology presented in this work allows for the rapid fabrication of highly conductive graphene substrates with high conductivity. The applied indirect stimulation technology allows contactless interaction with the cell culture, resulting in great application possibilities, e.g., for the use of implants.

## Materials and methods

### Heterophasic ink and substrates preparation

**FIGURE 1:**
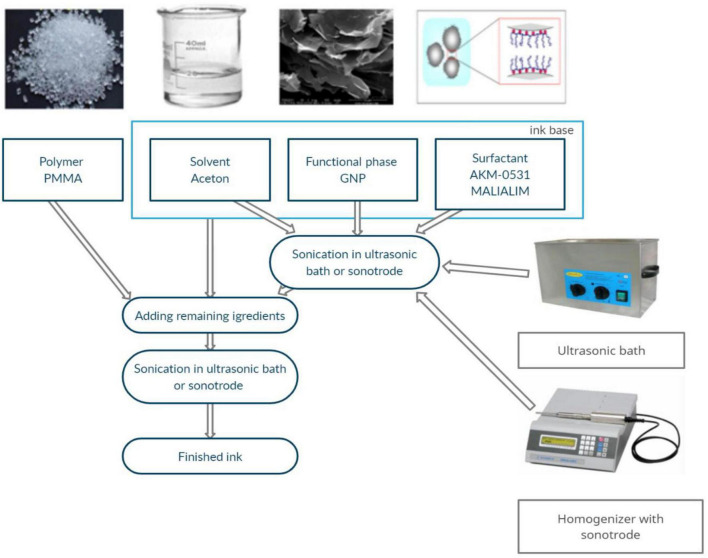
Graphene ink fabrication scheme (Dybowska-Sarapuk et al., 2020).

The ink production methodology and its components were presented in a previous paper (Dybowska-Sarapuk et al., 2018, 2020; Dybowska-Sarapuk, 2019). GNP M25 graphene nanoplatelets with an average particle size of 20–25 μm and a thickness of 19 graphene sheets produced by XG Science (Romanenko et al., 2006; Dybowska-Sarapuk et al., 2017, 2020) were used. The fabricated inks contained 0.5 wt. % GNP M25 graphene nanoplatelets. This amount of graphene nanoflakes provides high electrical conductivity and does not cause clogging the nozzle. An extended analysis of the subject matter was presented in a previous work (Dybowska-Sarapuk, 2019). The fabrication scheme of the graphene inks is shown in Figure 1.

The base ink was homogenised by ultrasonic energy using an InterSonic IS-10 ultrasonic scrubber (35 kHz operating frequency) and a Sonics Vibra Cell VC505 sonotrode homogeniser (maximum power 500 W and 20 kHz frequency). The baseline sonication process using the homogeniser lasted 1 min, while sonication using the scrubber lasted 30, 60, 90, or 120 min (Table 1). The beakers with the prepared ink were tightly covered with stretchable laboratory film to minimise the evaporation of acetone.

**TABLE 1:** Sonication methods and times (primary and secondary) of inks.

Used methods and times of ink sonication				
**Sonotrode**	**Ultrasonic bath**	**Ultrasonic bath**	**Ultrasonic bath**	**Ultrasonic bath**
1 min + 1 min	30 min + 15 min	60 min + 15 min	90 min + 15 min	120 min + 15 min

After combining the components with the pre-prepared base, the mix was sonicated again. In the case of inks homogenised using a sonotrode, the re-sonication time was 1 min, while the sonication time for inks in an ultrasonic washer was 15 min.

Two types of substrates were used in this study to test the conductivity: flexible Kapton^®^ polyamide film substrate (from Dupont™) with screen-printed silver contacts and polystyrene 6-well cell culture plates from Nest Scientific Biotechnology. The printed silver contacts allowed for a measurement of the resistivity of the layers. The dimensions of the tested layers were 24 mm × 24 mm in width and length. As in the previous study, coatings were applied via spraying. A manual Infinity Airbrush CR Plus Solo spray gun from Harder & Steenbeck was used. For Kapton film substrates, the produced layers were dried in a laboratory dryer at 120°C for 60 min (Dybowska-Sarapuk et al., 2018, 2020). For polystyrene substrates, a lower temperature of 60°C was required. Lowering the drying temperature increased the drying time to 300 min for the culture substrates.

Similar to the previous work, Rheological tests were performed using a Wells-Brookfield cone-plate viscometer model DV2T (cone type CP-40). A UNI-T laboratory multimeter, model UT804, was used to measure the resistance of the fabricated coatings. The thickness of the layers was measured with the DektakXT Bruker BNS stylus profilometer. Microscopic observations of layers were made using a Hitachi SU8230 scanning electron microscope (SEM) with an accelerating voltage of 5.0 kV. Images were taken with SEM at magnifications of 1 × 100 and 1 × 500.

### NE-4C cell culture and electrostimulation

Mouse NE-4C neural stem cells of neuroectodermal origin (ATCC Spontaneously immortalised cell line, Cat. # CRL-2925, RRID: CVCL_B063) were used to perform electrostimulation on graphene substrates. NE-4C neuroepithelial cell lines were obtained from brain follicles of 9-day-old mouse embryos. In this study, neural stem cells from 8 to 10 passages were used. The cells medium and culture substrate had been prepared, as described in detail in previous work (Dybowska-Sarapuk et al., 2020). Mouse NE-4C cells were seeded at 47,500 per well (5,000 per 1 *cm*^2^), which were then incubated at 37°C in an atmosphere of 5% *CO*_2_. Cells from the control group and stimulated with an electric field were cultured for 3 days.

A sinusoidal alternating current, a forcing frequency of 1 kHz, and voltage amplitudes of 5, 10, and 15 V were used for stimulation. After the stimulation, live cells were counted under a microscope using a Bürker chamber.

After culturing, the cells were fixed in 4% glutaraldehyde. Having been dehydrated and dried, the cell surface of the samples was covered with a thin layer of gold with a sputtering system. Microscopic observations were made using a SEM (Zeiss) with a SE secondary electron detector at an accelerating voltage of 5 kV. Specimens are observed in high vacuum and high resolution. Images were taken with an SEM microscope at 1 × 1,000 magnification.

## Results

### Rheology of graphene inks with different sonication methods and times

**FIGURE 2:**
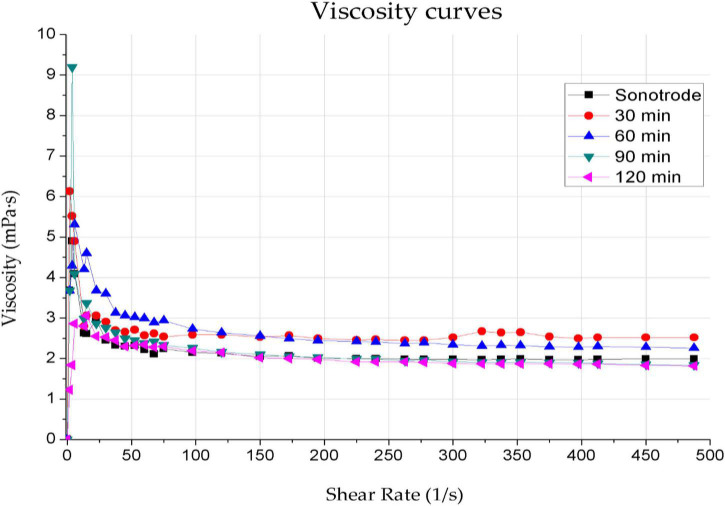
Dependence of viscosity on shear rate for graphene inks with sonication times of 30, 60, 90, and 120 min using an ultrasonic bath and 1 min using a sonotrode.

Inadequate ink viscosity results in uncontrolled spreading over the coated surface, resulting in a layer of non-uniform thickness. The composite’s viscosity depends on several factors, including the content and type of functional phase, the matrix material used and the solvent. Inappropriate viscosity degrades the mechanical and electrical properties of the graphene layer. Previous studies showed the particle size’s effect on solution viscosity and the effect of type and surfactant content on the rheological properties of inks. It also demonstrates that depending on the surfactant content, the viscosity values of the inks were in the range of 1.3–2.0 mPas. The viscosity dependence on shear rate for graphene inks with different sonication times and methods are shown in Figure 2, and the rheological measurements of graphene inks are shown in Table 2.

**TABLE 2:** Viscosity values for a shear rate of 487.5 mPas for graphene inks sonicated for 30, 60, 90, and 120 min using an ultrasonic bath and 1 min using a sonotrode.

Time and method of ink sonication	Viscosity value for the highest measured shear rate of 487.5 (mPas)
1 min, sonotrode	1.99
30 min, ultrasonic bath	2.52
60 min, ultrasonic bath	2.26
90 min, ultrasonic bath	1.84
120 min, ultrasonic bath	1.82

The viscosity curves of all the tested inks have the desired shape for spray-coating techniques, characteristic of shear-thinning non-Newtonian fluids. The curves represent a decrease in viscosity values as the shear rate increases, results from the gradual stacking of functional phase particles along the flow line. The viscosity decreases until no further increase in system ordering is possible – which corresponds to the Newtonian behaviour of systems at high shear rates (Kembłowski, 1973). Furthermore, the viscosity of the ink decreases slightly with increasing sonication time. The viscosity values obtained for the highest shear rate (487.5 mPas) are akin to the value of 2 mPas – they are in the range of 1.82–2.52 mPas. The sonication time only slightly influences the viscosity of the inks and does not affect the shape of the viscosity curves.

### Layer micro- and macro-geometry

**FIGURE 3:**
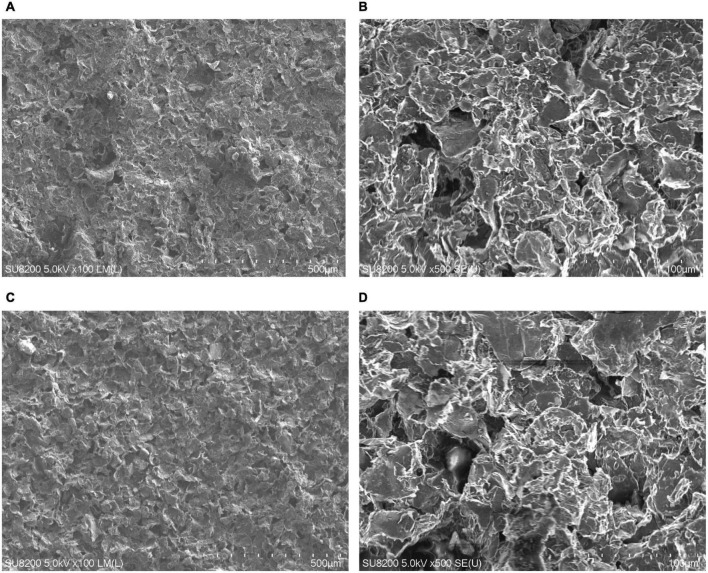
Scanning electron microscope images showing microgeometry of coatings. **(A,B)** Microgeometry of substrates made with inks sonicated using sonotrode for 1 min, **(C,D)** with inks prepared using the ultrasonic bath for 30 min. **(A)** One minute – sonotrode, ×100 magnification; **(B)** 1 min – sonotrode, ×500 magnification; **(C)** 30 min – ultrasonic bath, ×100 magnification; **(D)** 30 min – ultrasonic bath, ×500 magnification.

Graphene coatings, made using inks with different sonication times, were applied to flexible Kapton film substrates to determine the layer micro-geometry. The example images of graphene coatings on polystyrene substrates made using inks sonicated with a sonotrode are presented in Figures 3A,B, and a bath for 30 min in Figures 3C,D. SEM photographs of the microgeometry of the coatings revealed that no significant differences exist between the layers made using inks with different sonication methods and times. The fabricated layers are homogeneous with single, sparsely distributed clusters of particle agglomerates.

### Conductivity of graphene layers

Electrical conductivity is a crucial factor in using graphene substrates in the electrostimulation of nerve cells. As-low-as-possible resistance of conductive culture substrates is sought to increase the efficiency of stimulation and allow the use of a lower current signal to reduce potential cell weakness caused by the flow of an extensively strong electrical signal (Huang et al., 2019). Achieving high conductivity of coatings would be impossible without an appropriate ink homogenisation process, during which the functional phase particles are dispersed throughout the polymer matrix. Table 3 presents the measured resistivity values of the layers, with the measurement uncertainty of ±0.15 Ω. Table 4 shows the layers’ resistivity (24 mm × 24 mm).

**TABLE 3:** Results of coating resistance measurements prepared using inks with different sonication times (1 min – sonotrode, 30, 60, 90, and 120 min ultrasonic bath).

Resistance (Ω)				
**1 min – sonotrode**	**30 min – ultrasonic bath**	**60 min – ultrasonic bath**	**90 min – ultrasonic bath**	**120 min – ultrasonic bath**
45.21 ± 0.15	60.02 ± 0.15	136.56 ± 0.15	164.50 ± 0.15	90.43 ± 0.15

**TABLE 4:** Resistivity of the layers prepared using inks with different sonication times (1 min – sonotrode, 30, 60, 90, and 120 min ultrasonic bath).

Resistivity (Ω⋅m)				
**1 min – sonotrode**	**30 min – ultrasonic bath**	**60 min – ultrasonic bath**	**90 min – ultrasonic bath**	**120 min – ultrasonic bath**
1,623 ⋅ 10^–3^	2,154 ⋅ 10^–3^	4,902 ⋅ 10^–3^	5,905 ⋅ 10^–3^	3,246 ⋅ 10^–3^

To determine resistivity, the thickness of layers was measured using a profilometer. The average thickness of the coatings was 37.46 μm.

The measurements show a significant scattering of resistivity values for different sonication times. As the sonication time increases in the ultrasonic bath, a visible rise in coating resistance is present. The highest resistance values were obtained for inks sonicated for 90 and 120 min using the ultrasonic bath. On average, the resistance values of the coatings made with the inks sonicated for 120 min were twice as high as the resistance values achieved for the inks sonicated with the sonotrode. The lowest resistance values were obtained for the coatings made with the inks sonicated using a sonotrode and ultrasonic bath for 30 min; therefore, both were used for further electrostimulation studies.

### Electrostimulation results

**FIGURE 4:**
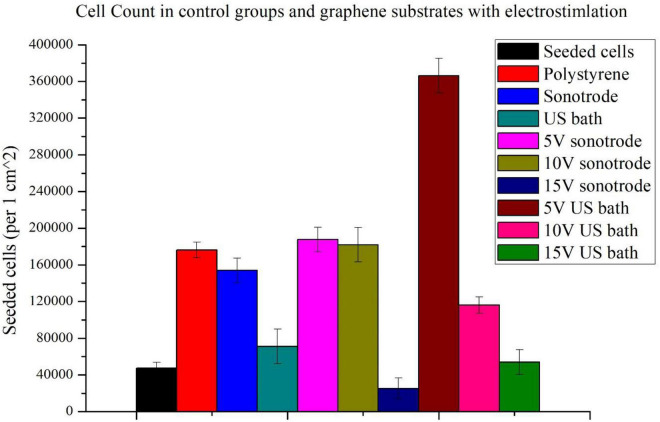
Comparison of cell counts: seeded, cultured on polystyrene substrate and graphene substrates made with inks sonicated using the sonotrode and ultrasonic bath with and without electrostimulation (5, 10, and 15 V).

**FIGURE 5:**
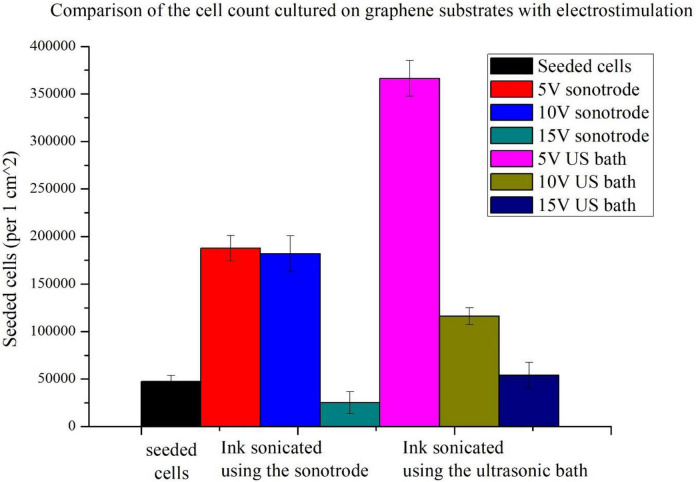
Comparison of cell counts cultured on graphene substrates and stimulated using different voltage amplitudes (5, 10, and 15 V).

To evaluate the effect of electrostimulation on NE-4C cells, the cell counts were examined on substrates without graphene coating, on graphene substrates without stimulation and with stimulation (Figure 4). To facilitate analysis, the results of the electrostimulation are shown separately (Figure 5). Figure 4 shows the comparison of cell counts from all culture groups: polystyrene culture plate without graphene layer, with graphene layers (ink sonicated with the sonotrode and ultrasonic bath, named sonotrode and US bath on figures, respectively) without stimulation; and cultures on graphene with electrostimulation with the voltage amplitudes of 5, 10, and 15 V. The initial number of seeded cells (47,500 cells per 1 *cm*^2^) is also included in the graph (red column – seeded cells).

Firstly, it can be seen that the use of graphene substrates significantly increases cell counts. The use of graphene substrates alone increases cell counts more than threefold. When applying graphene substrates, cell counts approximating those on polystyrene control substrate without graphene coating were obtained. A high increase in cell number is observable for the graphene substrate prepared with ink sonicated using a sonotrode.

For both types of substrates, the highest population size was obtained for cells stimulated with the lowest voltage amplitude of 5 V. More than the sevenfold increase of the cell count (compared to seeded cells) was obtained using 5 V electrostimulation and graphene substrate prepared using the ink sonicated in the ultrasonic bath. In the case of the other substrate, an almost fourfold increase in cell count was obtained. Slightly lower cell counts were obtained with a stimulation voltage of 10 V. The weakest stimulation effects, for both types of substrates, were obtained using the highest voltage amplitude of 15 V. The 15 V sonotrode substrate was the only one in which the decrease in cell count was observed. In the 15 V US bath substrate, the cell count only slightly higher than the number of seeded cells was observed.

**FIGURE 6:**
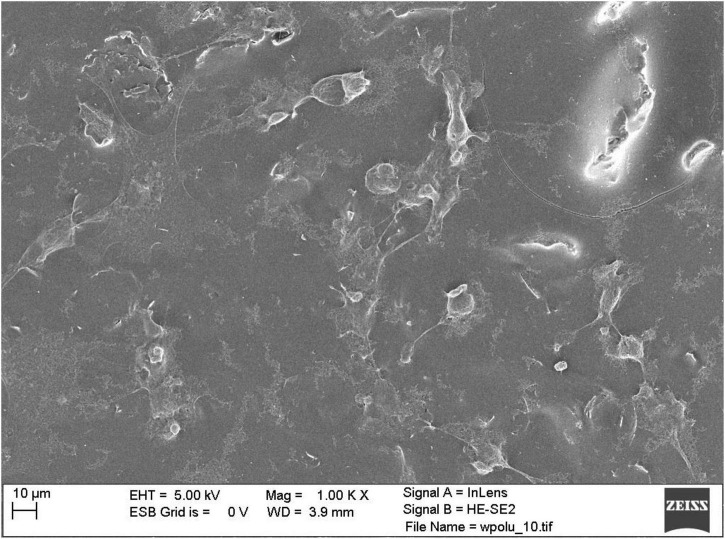
Scanning electron microscope photograph of control group neural stem cells on graphene layer without stimulation.

**FIGURE 7:**
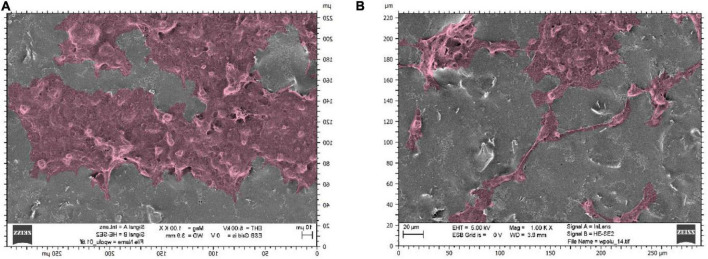
Scanning electron microscope photographs showing neural stem cells on graphene layers stimulated with a voltage of 5 V amplitude: substrates made with sonicated inks using **(A)** sonotrode **(B)** ultrasonic bath.

Observations of selected culture substrates were also made using SEM microscopy to assess the effect of electrostimulation. Figure 6 shows example images of graphene substrates along with embedded neural stem cells from the control culture. Figure 7 shows the cells after electrostimulation with a stimulating voltage of 5 V amplitude, with cell clusters marked in colour to facilitate the analysis of photographs.

The cell count study and their microscopic observations confirmed the positive effect of graphene substrates and electrostimulation on neural stem cell behaviour. Using graphene substrates increases cell counts by 300% (without electrostimulation) and over 700% by using 5 V stimulation. Graphene layers improve the shape and adhesion of cells to the substrate. It is clear that lower values of stimulation voltage amplitude improve the cell behaviour. The highest cell count for both types of substrates was obtained when electrostimulation with a voltage amplitude of 5 V was applied. Therefore, these substrates were selected for further observations using SEM microscopy.

## Discussion

The literature analysis of graphene surface application evidences a high demand for innovative solutions in the field of neural tissue engineering. The production of a biocompatible layer supporting the development of nerve cells, promoting their adhesion and proliferation will allow for significant improvements in the treatment of many such neurodegenerative diseases as neural tube defects, spinal cord injuries, or neuropathies. Achieving the desired homogeneity of the produced composite is crucial for the substrate’s conductivity, which makes them useful in the electrostimulation of nerve cells. The properties of graphene layers are also affected by the composite fabrication process itself particularly the time and method of solution homogenisation.

### Ink rheology

The studies showed a significant influence of the graphene ink preparation method on the properties of the graphene coatings. Here, two alternative ultrasound sonication methods were used, which allowed for high dispersion of graphene particles, resulting in homogeneous, highly conductive graphene layers successfully used in the electrostimulation of neural stem cells.

While comparable ink viscosity values decreased with the increasing homogenisation time. Many factors can influence the rheological properties of fluids, including the particle’s shape and size, the molecular weight of the functional phase or particle size distribution in solution (Wilczynski, 2001, Nguyen et al., 2007). The viscosity of the solution increases as the molecular weight rise and decreases as its distribution expands (Wilczynski, 2001). In prepared heterophasic inks, the increased ultrasound sonication time presumably could result in a fragmentation of the particles in the solution. The resulting increase in particle size distribution according to the literature reports, increases in particle size distribution lead to a decrease in ink viscosity.

Despite the lack of differences in ink viscosity, apparent differences were observed in the conductivity of layers produced at different sonication times. As the viscosity decreased (with longer sonication times), the conductivity of the layer decreased. This is presumably related to the degree of graphene particle dispersion in the produced inks. The sonication time probably affected the fragmentation of graphene nanoplatelets and also changed the degree of the graphene particles’ surroundings by the surfactant, thus influencing the formation of particle agglomerates. According to Dziubiński et al. (2014), if the suspension contains in its composition a significant fraction of small particles, they tend to be surrounded by larger particles, which reduces the interactions between them. In the studied inks, the reduction of functional phase interactions is associated with a decrease in viscosity and a deterioration of electrical properties. However, analysing the relationship between viscosity and conductivity of the layers requires a more detailed examination and additional studies – among others, of the size of graphene nanoplatelets obtained in ink after the sonication process.

Nevertheless, each manufactured ink fulfils the rheological requirements enabling its application in the spray coating technology. The sonication time and method did not cause significant discrepancies in the viscosity values of the inks. It can be concluded that both sonication methods are effective ways to homogenise solutions. The relationship between viscosity and conductivity of the layers requires further investigation and additional studies – including the size of graphene nanoplatelets obtained in ink after the sonication process.

### Microgeometry of the layers

Observations of the micro and macro geometries of the substrates conclude that both sonication methods can be successfully used in the homogenisation of graphene inks. However, the taken photographs do not allow an assessment of the effect of sonication time on the dimensions of the graphene particles themselves. Particle dimensions are an essential parameter for coating applications in tissue engineering, as they could strongly influence the occurrence of cytotoxic phenomena. Therefore, in future work, it is necessary to perform SEM photography with higher accuracy to measure the size of the flakes.

### Conductivity of the layers

The lowest resistivity values were obtained for the coatings made with the ink sonicated using a sonotrode for 1 min and an ultrasonic bath for 30 min. The obtained resistivity of the films were 1,623 ⋅ 10^–3^ Ω⋅m and 2,154 ⋅ 10^–3^Ω ⋅m, respectively, for sonotrode and ultrasonic bath. The results show that a short, 1-min sonication process using a homogeniser or extending the process to 30 min when using an ultrasonic bath is optimal for the accurate combination of surfactant particles with graphene flakes. The study showed that the obtained resistance values strongly depend on the graphene ink’s method and sonication time. With an extension of the sonication time, apparent increase in the resistance of the coatings can be seen. This phenomenon may be due to the excessive fragmentation of graphene nanoplatelets, as flakes with smaller dimensions are weaker conductors.

Through the successful use of a sonotrode, the efficiency of the fabrication process of graphene heterophasic inks was increased by shortening the sonication time from 90 to 2 min while increasing the conductivity of the layers. Comparing the fabrication times of the inks made in our previous work (90 + 15 min sonication) (Dybowska-Sarapuk et al., 2020), one can estimate a more than the decimal reduction in the fabrication process of inks while maintaining the appropriate rheological properties of the ink and conductivity of the coatings.

### Electrostimulation effects

Upon analysing the results, graphene substrates and electrostimulation significantly increase the cell counts. The simulations carried out in this work, using different stimulating voltages with amplitudes, showed over a 700%-cell-count increase when 5 V was applied. The produced substrates improved cell adhesion and morphology, as shown in the SEM photographs in the section below. The fabricated graphene substrates had a beneficial effect not only on cell adhesion but also on cell proliferation. Using graphene substrates enabled to increase cell count by 300% (without electrostimulation).

The occurrence of functional groups on graphene surfaces could influence the types of cell surface receptors and proteins, such as the cellular adhesion molecules (CAM), which cells use to bind to graphene surfaces (Chen et al., 2012; Lee et al., 2015). Lee et al. (2015) confirm the expression of neural CAM (NCAM) is significantly increased by exposing cells to an electromagnetic field and graphene substrate. Authors claim that despite using the low-frequency electromagnetic field, the current flow generated by the electromagnetic field can alter the neuronal membrane potential, thereby leading to the activation of intracellular signalling, enhanced cell adhesion and differentiation (Lee et al., 2015).

Cell adhesion plays a significant role in regulating such processes as growth, proliferation and migration. The increase in the number of cells on graphene illustrates a positive effect on cell adhesion, also confirmed by Hong et al. (2014). Moreover, Lee et al. (2015) confirm that the expression of NCAM, an adhesion molecule secreted by cells, can be significantly increased by exposing cells to an electromagnetic field and graphene substrate. Despite the low frequency, the current flow generated by the electromagnetic field can alter the neuronal membrane potential, thereby leading to the activation of intracellular signalling and enhanced cell adhesion and differentiation.

For both types of substrates, a decrease in cell count is observed as the stimulation voltage amplitude increases. This observation indicates that the stimulation voltage amplitude of 15 V is too high and probably hinders the cells from adhering correctly to the substrate, thus preventing their proliferation, which is also stated by Lee et al. (2015). The obtained decrease in the cell count may depend on many factors, such as a possible fragmentation of the particles caused by sonication using a sonotrode, an inaccurate combination of ink components, a too thick or too thin graphene layer or a weakening of a particular cell group.

In the study, the 5 V voltage amplitude was used. However, the literature reports confirm the efficiency of even lower stimulating voltages (Heo et al., 2011; Das et al., 2017; Huang and Zhu, 2019; Uz et al., 2020). This issue will be addressed in subsequent research. Moreover, the complex relationship between neural stem cell and graphene layer properties needs further investigation and exploration. Similarly, preparing substrates for microscopic observation also needs further investigation to define their effects on cellular features such as shape, size and cell network formation. The obtained results align with those in other studies (Lee et al., 2015) provide a foundation for further research on approximating the regeneration of nervous tissue with graphene substrates and electrostimulation.

## Conclusion

In conclusion, we can state that using graphene substrates as cellular scaffolds enhance and improve neural tissue regeneration protocols. The preliminary data showed the efficiency of the proposed simulation method and the beneficial effects of graphene substrates and electrical stimulation. We propose an alternative tissue stimulation method, which could be a starting point for new, innovative treatment routes. The high homogeneity and conductivity of the layers allowed them to be effectively used in the electrostimulation of neuronal stem cells. The increased cell count of cells cultured of the graphene substrates indicates that our substrates provide an environment suitable for cells’ development, mainly supporting cell adhesion, which enables their proper growth and proliferation. Graphene substrates show significant potential in cellular scaffolds and regenerative medicine, which in the future may contribute to the development of effective clinical therapy for peripheral nerve injuries.

## Data availability statement

The original contributions presented in this study are included in the article/supplementary material, further inquiries can be directed to the corresponding author.

## Author contributions

ŁD-S: conceptualisation and methodology. WS: writing—first draft, experiments—materials preparation and spray coating, and figures preparation. AG: experiments—cell culture. JK: formal analysis. WS and JK: writing—review and editing. ŁD-S and MJ: supervision and funding acquisition. All authors contributed to the article and approved the submitted version.
